# Supplementary material for: Demonstrating the processes and outcomes of a rural Community Mental Health Rehabilitation Service: A realist evaluation
Source: PLoS One. 2021 Nov 23;16(11):e0260250. doi: 10.1371/journal.pone.0260250 (PMC8610260; doi:10.1371/journal.pone.0260250)
Supplement: S1 File — (DOCX) [file pone.0260250.s001.docx]

**Supplementary File 1: Service Eligibility and Consumer Profile**

**Eligibility**

Consumers with an enduring moderate to severe mental illness with identified rehabilitation needs that cannot be addressed through less restrictive service options.

Suitable consumers may present with the following:

- 18+
- Consumers who are able to engage in a personalised rehabilitation process
- Have an identified discharge address
- Must be a current consumer of a community mental health service
- Consumers who would benefit from a structured environment and intensive therapeutic program
- Identified rehabilitation goals related to difficulties in their personal functioning
- Mental illness is the primary diagnosis

**Consumer profile**

The service is aimed at supporting people with a primary diagnosis of mental illness who have high and complex needs and with some or all of the following features:

- Significant functional disabilities are indicated in the areas of life or social skills and self-care resulting in significant rehabilitation needs.
- Would benefit from intensive rehabilitation management due to significant impact of their mental illness.
- Would benefit from living in a supported residential environment to assist in returning to achievable and sustainable level of independent living.
- A person’s need cannot be met by a less restrictive option, or trials of periods of less intensive community support have not been able to meet the consumer’s needs.
- The person does not pose a significant risk to themselves or others.
- Be willing to live in a shared living environment (if required, due to single accommodation not being available).
- Be willing to participate in a planned rehabilitation support program.

Individuals are likely to have one or a combination of the following characteristics:

- Unremitting symptoms resulting from mental illness.
- Have not succeeded at independent community living previously.
- Risk factors including complex health (medical and allied health), lifestyle and/or behavioural needs and co morbidities.
- Patterns of difficulty in engaging with services creating risk to self and/or others.
- Lack of natural support from family/friends and/or connectedness with the community.
- Significant grief and loss issues impacting on long term mental health.
- May have a complex trauma history including experience with interpersonal violence, adversity, and exposure to trauma over extended periods of time.

Participation in the service is voluntary. It is acknowledged that some people will, at times, need supportive encouragement to engage with the CMHRS program until they feel safe and secure. Homelessness, experience with the criminal justice system, orders under the *Guardianship and Administration Act (1993*), *Mental Health Act (2009)* and/or the *Criminal Law Consolidation Act: Mental Impairment Provisions* do not impact on eligibility.
