# Supplementary material for: Demonstrating the processes and outcomes of a rural Community Mental Health Rehabilitation Service: A realist evaluation
Source: PLoS One. 2021 Nov 23;16(11):e0260250. doi: 10.1371/journal.pone.0260250 (PMC8610260; doi:10.1371/journal.pone.0260250)
Supplement: S2 File — (DOCX) [file pone.0260250.s002.docx]

**Supplementary File 1 Interview/Focus Group Guides – Consumer and Staff**

**Consumer Guide**

Researcher introduction

**Hi my name is ….**

**Thank you for agreeing to meet with me and share your experience of the Community Mental Health Rehabilitation Service.**

**I have some questions to help guide our conversation which I will refer to if that is ok with you?**

**Plus, you are welcome to share anything that you think is relevant to help us understand how the service is helping consumers and also if there are ways it can be improved.**

**If at any time you feel uncomfortable and want to pause or stop the interview, please let me know. And if you feel any distress please let me know, we can stop the interview at any time and arrange support for you.**

**Let’s start by thinking about the structure and program. During your time at CMRHS you would have spent time with individual staff – 1:1 sessions, participated in groups and had unstructured time where you could do your own thing.**

**Can you tell me about the time you spent 1:1 with a staff member?**

Follow up probe questions if needed:

*Structure*

- How often would you spend time with staff?
- How much time would you have 1:1 with staff?
- Did you have a choice of when 1:1 conversations occurred?
- Were 1:1 sessions planned or ad hoc?
- Did you prefer planned or unplanned 1:1?
- If you wanted 1:1 time was this available within a reasonable period?

*Scope of intervention/ service*

- What sort of things did you do in your 1:1 time with staff?
- To what extent were your needs meet during the 1:1 sessions?
- How did the discussions in your 1:1 time relate to your goals about recovery?
- To what extent did the 1:1 sessions meet your expectations?
- Do you feel the amount of 1:1 intervention available was enough for you?

**How about the groups? Can you describe them to me and how often you were involved in them?**

Follow up probe questions if needed:

*Structure*

- Did you feel that this were enough groups? Or too few?
- Were there particular groups you enjoyed?
- What was it about the groups you enjoyed?
- What didn’t you like about the groups?
- Did you have a preference for the type of group offered?
- How often were you offered psychoeducational groups? (Clarify which groups this were if necessary)
- Did the location of the groups impact on your engagement?
  - If so, what was useful and what was not useful?
- Was the amount of group work available enough for you?

*Scope of intervention/ service*

- Can you share with me the type of things you did in the groups?
- To what extent did the group sessions meet your recovery needs?
- In what ways did the group sessions relate to your goals about recovery?
- Did what was offered in the groups meet your expectations?
- Where there particular topics that your found particularly helpful, if so what where they?

**How did you find the unstructured time? What sort of things did you do?**

Follow up probe questions if needed:

*Structure*

- How often did you have time to yourself?
- What do you think of the balance between rehabilitation activities and free time?
- Were there things to do in your free time?
- How did you find having free time?

*Scope of intervention/ service*

- In what ways did the activities planned without staff relate to your recovery?
- How did the available resources facilitate engagement in activities when staff were not around?
- Looking back what do you think is a good way people to spend their free time at CMHRS?
  - Did you do these activities, why/why not?

**I would like to ask you about the information you received before coming to CMHRS and get a sense of how helpful it was. Can you tell me what you remember about the information you were provided with?**

Follow up probe questions if needed:

- Was the information offered sufficient? If not, what could/should have been offered?
- What part of the orientation was useful/not useful?
- Is there anything you feel is really important to know before transitioning to CMHRS?

**Having explored how you were prepared for participating in CMHRS I’d like to explore the supports and structures around discharge. How was the discharge process for you?**

Follow up probe questions if needed:

- What information were you provided with in relation to your discharge?
- Was the information sufficient? If not what else would have been helpful?
- In terms of the discharge process what were the most and least helpful aspects?

**Thank you – all this information is really helpful, and we only have a few more questions to go.**

**This next question is trying to find out the extent to which participation in the CMHRS has impacted on your recovery journey. So, can you share with me what you feel has impacted on your recovery journey the most?**

Follow up probe questions if needed:

- What helped your recovery?
- What hindered your recovery?
- Where there specific interventions that made a difference for you?
- What was it about that particular intervention that worked well?
- What do you feel you have learnt from participating in the CMHRS?
- Has it influenced your confidence in your ability to do things?
- Do you feel more confident in looking after yourself?
- What sort of changes have you seen in yourself?
- What changes do you think others have seen in you?

**On reflection to what extent do you feel you achieved what you hoped for?**

**To what extent do you feel as if you had a say on your rehabilitation process?**

**Can you think of any ways in which the service could be improved?**

Follow up probe questions if needed:

- If you were running the service what if anything would you change?

**Finally, would you recommend the service to someone else?**

**Thank you for spending time with me today and sharing your perspectives on the CMHRS – your feedback will help with ongoing quality improvements. I feel privileged to have had a chance to hear your recovery story.**

**Staff Focus Group/Interview Guide**

**Hi my name is ….**

**Thank you for agreeing to meet with me and share your experience of the Community Mental Health Rehabilitation Service.**

**The purpose of the focus group is to explore how the service operates including perceptions of the overall impact of the service for consumers, what components of the service and service structures are believed to benefit consumers and staff and exploration of how the service can continued to be developed**

**Before we start it is important to discuss some key considerations for the focus groups**

**Firstly, it is important that the conversation that we have today remains confidential (within this group)**

**Secondly, people in the group should only discuss/disclose information that they feel comfortable to share. If there is something that you want to share but do not feel comfortable to share in the focus group, please let me know privately and I will organise a time to meeting with you individually.**

**I have some questions to help guide our conversation which I will refer to if that is ok with you?**

**Plus, you are welcome to share anything that you think is relevant to help us understand how the service is helping consumers and also if there are ways it can be improved.**

**Let’s start by getting you to reflect on the 1:1 time you spend with consumers at CMHRS.**

**1:1 interaction with consumers**

**Can you tell me about the type of 1:1 interaction you have with consumers?**

Follow up probe questions if needed:

Structure:

- On a typical shift how often would you spend time with consumers?
- How long would a typical session with consumers go for?
- How often would 1:1 sessions planned/structured or adhoc?
  - What would be the shortest/longest?
- Are there any differences in planned/ad hoc interactions?
- What do you feel are the strengths and challenges of planned or unplanned interactions?
- If consumers request 1:1 time do you normally have the flexibility to respond?
- Can you tell me about the amount of direct client contact vs indirect client contact?
  - If we were to talk about percentages what would these be?
- Do you have the resources/time/support to plan your interactions the way you would like to?
- Do you feel the amount of 1:1 intervention we offer is enough?
  - Frequency and duration
- If a consumer requests unplanned support, how often are you able to facilitate it?
  - Immediately, that shift, later in the week.

**Reflecting on the 1:1 service that you provide what facilitates or hinders what you do?**

*Scope of intervention/ service*

- What typical interventions/tasks would you do in your 1:1 consumer interactions?
- How often do typical interventions that we offer as a service meet the individual need?
- Are planned interactions reflective of care plans/rehabilitation goals?
- Are there interventions that we are unable to provide?
  - What barriers do we feel prevent us from particular interventions?
- How often would interactions run according to plan?
  - Time, type of intervention, immediate needs, crisis, flexibility, available.
  - Frequency and duration

**Group interaction with consumers**

Ok, now can I get you to reflect on the groups provided at the CMHRS and unpack the decision making involved in offering and delivering groups and what has worked and what hasn’t? Starting with the following question:

**Can you describe the groups currently offered and how staff are involved?**

Follow up probe questions if needed:

*Structure*

- Do we feel there are enough groups offered? Or too few? Too many?
- How often do we offer groups?
- Do the location of the groups impact on your engagement?
  - If so, what was useful and what was not useful?
- How are groups planned?
- Do you have a preference to how groups are planned?
- What would stop you from running a planned group?
  - Staffing, confidence, no consumers, weather, room not available

**Reflecting on the groups offered what facilitates or hinders these?**

*Scope of intervention/ service*

- Can you share with me the type/content of groups offered?
- To what extent did the group sessions support consumer recovery?
- Did what was offered in the groups meet consumers expectations?
- Where there particular topics that you feel are particularly helpful for consumers, if so what where they?
- Do you have a preference for the type of group offered?
- Are they any groups that we are unable to provide?
  - If so, what are they, what are the barriers?

**Consumer unstructured time**

Now we would like you to consider consumers’ unstructured time

**How do consumers use their unstructured time?**

Follow up probe questions if needed:

*Structure*

- How often do consumers have time to themselves?
- Do we feel the balance between rehabilitation activities and free time is adequate?
- How would support consumers to plan their free time?
- What are the barriers/enablers to using free time in a way that aids consumer recover?
- How did the available resources facilitate engagement in activities when you were not around?
  - Are there enough resources to facilitate independent engagement in activities?

**What suggestions do you have to facilitate the use of unstructured time to aid consumer recovery?**

*Scope of intervention/ service*

- In what ways did the activities planned without staff relate to consumer recovery?
- Looking back what do you think is a good way for consumers to spend their free time at CMHRS?
  - Do consumers often participate in these activities, why/why not?

**Discharge**

We would now like to explore the supports and structures around discharge.

**What is the current discharge process for consumers?**

Follow up probe questions if needed:

- What information are consumers provided with in relation to their discharge?
- How do we come to a decision about a consumer’s discharge?
- Let’s talk about planned vs unplanned discharges
  - Is the process different? What does support look like? What do you think about unplanned discharges? What are the common reasons for unplanned discharges?
- Do we feel the information we provide about discharge is enough? If not what else could be helpful?
- In terms of the discharge process what are the most and least helpful aspects?

**Thank you – all this information is really helpful, and we only have a few more questions to go.**

**Impact of the CMHRS on consumers recovery**

The next questions will explore the extent to which participation in the CMHRS impacts on consumer recovery journey.

**Can you share with me what you feel has impacted on the consumers recovery journey the most?**

Follow up probe questions if needed:

- What supports recovery?
- What hindered recovery?
- Were there specific interventions that made the biggest difference for consumers?
  - What was it about that particular intervention that worked well?
- What do you feel you consumers learn from participating in the CMHRS?
- Do consumers leave feeling confident in their ability to do things?
  - Such as?
- Do you feel more confident in looking after yourself?
- What sort of changes do we/family members/care co-ordinators see in consumers?

How do we feel the CMHRS impacts on the 4 quadrants of recovery: Mastering my illness, looking forward, doing things I value, connecting and belonging?

**On reflection to what extent do you feel CMHRS achieves recovery for consumers?**

Follow up probe questions if needed:

To what extent do you feel as if you have an influence on consumers rehabilitation?

**Can you think of any ways in which the service could be improved?**

Follow up probe questions if needed:

- What if anything would you change to better assist the recovery of consumer?
- What if anything would you change to improve the work environment for staff?

**Conclusion**

**Do you have any other comments that may assist with the evaluation of the service?**

**Thank you for spending time with me today and sharing your perspectives on the CMHRS – your feedback will help with ongoing quality improvements.**
